# Multilocus sequence typing characterizes diversity of *Ureaplasma diversum* strains, and intra-species variability induces different immune response profiles

**DOI:** 10.1186/s12917-020-02380-w

**Published:** 2020-05-26

**Authors:** Yasmin M. F. S. Andrade, Manoel N. Santos-Junior, Izadora S. Rezende, Maysa S. Barbosa, Aline T. Amorim, Ícaro B. S. Silva, Ellunny C. Queiroz, Bruno L. Bastos, Guilherme B. Campos, Jorge Timenetsky, Lucas M. Marques

**Affiliations:** 1 Universidade Estadual de Santa Cruz, Brazil, Jorge Amado Highway, Km 16, Salobrinho, Ilheus, Bahia 45662-900 Brazil; 2 Instituto Gonçalo Muniz, Fundação Oswaldo Cruz, Salvador, Brazil, Waldemar Falcao Street, 121, Candeal, Salvador, Bahia 40296-710 Brazil; 3 Instituto de Ciências Biomedicas, Universidade de Sao Paulo, Brazil, Professor Lineu Prestes Avenue, 2415, Butantã, São Paulo 05508-900 Brazil; 4 Instituto Multidisciplinar em Saúde, Universidade Federal da Bahia, Brazil, Hormindo Barros Street, 58, Candeias, Vitória da Conquista, Bahia 45029-094 Brazil

**Keywords:** *Mollicutes*, Genetic diversity, Sequence type, Clonal complex, Gene expression, Cytokines

## Abstract

**Background:**

*Ureaplasma diversum* is a pathogen found in the genital tract of cattle and associated with genital disorders such as infertility, placentitis, abortion, birth of weak calves, low sperm motility, seminal vesiculitis and epididymitis. There are few studies evaluating the genetic diversity of *U. diversum* strains and their influence on the immune response in cattle. Therefore, to better understand genetic relationships of the pathogenicity of *U. diversum*, a multilocus sequence typing (MLST) scheme was performed to characterize the ATCC 49782 strain and another 40 isolates recovered from different Brazilian states.

**Results:**

Primers were designed for housekeeping genes *ftsH, polC, rpL22, rpoB, valS* and *ureA* and for virulence genes, phospholipase D (*pld*), triacylglycerol lipase (*tgl*), hemolysin (*hlyA*), MIB-MIP system (*mib,mip*), MBA (*mba*), VsA (*VsA*) and ribose transporter (*tABC*). PCRs were performed and the targeted gene products were purified and sequenced. Sequence types (STs), and clonal complexes (CCs) were assigned and the phylogenetic relationship was also evaluated. Thus, a total of 19 STs and 4 CCs were studied. Following the molecular analysis, six isolates of *U. diversum* were selected, inoculated into bovine monocyte/macrophage culture and evaluated for gene expression of the cytokines TNF-α, IL-1, IL-6, IL-10 and IL-17. Differences were detected in the induction of cytokines, especially between isolates 198 and BA78, promoted inflammatory and anti-inflammatory profiles, respectively, and they also differed in virulence factors.

**Conclusion:**

It was observed that intra-species variability between isolates of *U. diversum* can induce variations of virulent determinants and, consequently, modulate the expression of the triggered immune response.

## Background

*Ureaplasma diversum*, the only ureaplasma found in cattle, was first isolated in 1967 and produces urease and hydrolyzes urea in ammonia, and has no cell wall or mobility structures [1]. It invades epithelial cells of the respiratory and genital tracts from cattle, and therefore, is related to reproductive diseases by interfering with implantation and embryonic development [2]. Disorders such as vulvovaginitis, salpingitis, endometritis, fetal alveolitis, infertility, abortion or birth of weak calves in cows and seminal vesiculitis, epididymitis in bulls, are strongly associated with *U. diversum* [2]*.* Few studies have reported the mechanisms of *U. diversum* pathogenicity; however, it is known that ammonia, its main metabolite, is toxic to cells and tissues of the host it invades [3]. In addition, bacterial phospholipase production favors prostaglandin synthesis, promoting uterine contractions and abortions [3]. The infection also reduces reproductive potential by inducing inflammatory cytokine secretion by macrophages [3]. *Ureplasma diversum* has been demonstrated to be able to invade and induce apoptosis in HEp-2 cells, invade sperm leading to loss of semen quality, as well as express molecules that trigger immune response by proinflammatory cytokine production (IL-1α, IL-1β, IL-6, IL-8 and TNF-α), opsonization by antibodies and complement system, and infiltration of neutrophils and macrophages in infected sites [4–6].

*Ureaplasma diversum* isolates may present distinct patterns of virulence, pathogenicity and gene expression that trigger the immune response [7]. In this context, molecular typing techniques help better understand the diversity in this mollicute. Multilocus sequence typing (MLST) is an advantageous and sensitive technique for assessing genetic variability by comparing sequences from a set of constitutive genes and precisely identifying nucleotide changes, in addition to accommodating information in a database [8]. Thus, the present study aims to observe the diversity of strains isolated in Brazil and to evaluate whether the variability can result in differences in expressing immunological markers, contributing to new perspectives on interventions in preventing and treating diseases caused by *U. diversum*.

## Results

### MLST scheme characterized the diversity of strains of *U. diversum*

**Fig. 1:**
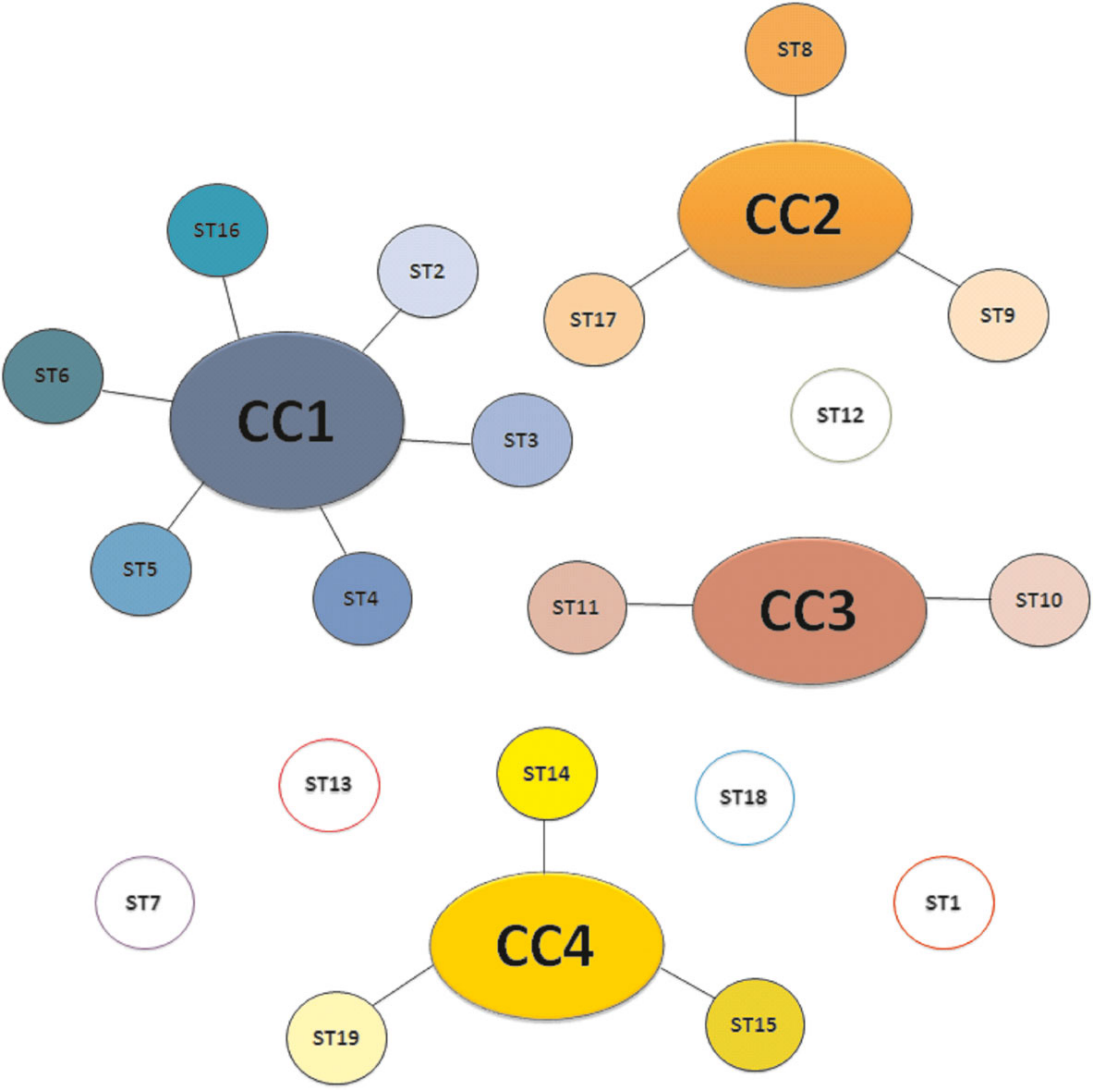
Schematic model MLST. Each large circle represents a CC linked with respective STs, in smaller circles. Circles not linked to any CC are singleton STs

In this study, seven housekeeping genes of *U. diversum* were selected; however, only six were sequenced successfully and *rpL22* gene did not show polymorphisms between the isolates. The size of the amplicons varied between 248 bp and 809 bp (Table 1). NRDB assigned the respective alleles from the identification of variations between the sequences, and the allelic profile formed for each strain defined the STs (Table 2). Hence, the 45 isolates were classified into 19 STs, and some STs were grouped into 4 CCs. Six STs were assigned to CC1, 3 STs grouped in CC2 and CC4, 2 STs in CC3 while 5 STs were not grouped in any CC (Table 3).Thus, 26 isolates (57.7%) were present in different CCs, and 19 isolates (42.3%) were not grouped. Therefore, genetic variations were observed among the 45 strains and between farms (Table 3). Figure 1 outlines these different clonal groups with their respective STs, as well as unique STs.

**Table 1:** Genes selected for MLST scheme and virulence analysis, with their respective primers designed from access to categorized genes of *U. diversum* ATCC 49782. F indicates forward and R indicates reverse

Gene	Primer	Primer Sequence 5′ – 3’	Gene length	Amplicon
MLST scheme				
*ftsH*	ftsH - F	CAGTGCGTGATAACAAAACTG	1533 bp	753 bp
	ftsH - R	CAACTACATCAGCTTCTTCAGC		
*polC*	polC – F	ACGGATCTCAGGTTTATCTCAT	4305 bp	653 bp
	polC – R	CAGGAAGCATTGTAAAAGGTG		
*rpL22*	rpL22 – F	CTCAGCAGTTGCTAATGTAACT	972 bp	809 bp
	rpL22 – R	AGTATTGGAACCCACTTGG		
*rpoB*	rpoB – F	AGCCGTATGAACATTGGAC	4281 bp	718 bp
	rpoB – R	ACTTGAATGATTCAGGCATTCC		
*ureA*	ureA – F	CGACCTTGCTCGTAGACGT	306 bp	248 bp
	ureA – R	AGATAGGTTCGTGAATTGACAC		
*valS*	valS – F	TGCTCCTTGTATTAGTGAAGAC	2616 bp	468 bp
	valS – R	GCTGGGGCTTTAGATGTAAA		
Virulence genes				
*pld*	pld – F	CCAACTGAAGAGTTGATTGTAGC	615 bp	396 bp
	pld – R	GGTTTCAACAACCTGTCTTGCG		
*tgl*	tgl – F	ATTGCTTGTATGCTTGCGGAAG	873 bp	546 bp
	tgl – R	ATCACCCTCTGATTCTTCAACT		
*hlyA*	hlyA – F	GGAGTTGTTCGTTATGTTGGA	1389 bp	463 bp
	hlyA – R	CAACAAGCCGACTAAATCCAGT		
*mib*	mib – F	GATACACCGATTGAACCTCCA	2484 bp	924 bp
	mib – R	TCAACCAGGATAACTTCCTTGA		
*mip*	mip – F	ACGAATTTGAAGGTAAGGTAGC	2400 bp	539 bp
	mip – R	TCAACTTGCTTGTTGACCAGG		
*mba*	mba – F	GTTGGTGCTATTATGGCAGGG	1398 bp	704 bp
	mba – R	CCATTGTTTGGACTAGGAGTT		
*VsA*	VsA – F	AACCTAATCTTGGTTCTGACC	1227 bp	255 bp
	VsA – R	CTTCGCTTCCTGAACCTTCCAT		
*tABC*	tABC – F	CGCTTTATTTGCTGCGGTATTA	936 bp	683 bp
	tABC – R	CAAGTGCTAAGAATCCAGCTCC

**Table 2:** Allelic profile and sequence types (ST) of the 45 strains of *U. diversum*

Strains	Allelic profile						ST
	***ftsH***	***polC***	***rpL22***	***rpoB***	***ureA***	***valS***	
5 T	1	1	1	1	1	1	1
7 T	1	1	1	1	1	1	1
10 T	1	1	1	1	1	1	1
13 T	1	1	1	1	1	1	1
16 T	1	1	1	1	1	1	1
34	2	2	1	2	1	2	2
35	2	2	1	3	1	2	3
37	2	2	1	3	1	2	3
47	2	2	1	3	1	2	3
51	2	2	1	3	1	2	3
52	3	3	1	2	1	3	4
54	3	3	1	4	1	3	5
55	3	3	1	4	1	3	5
56	4	3	1	4	1	3	6
59	4	3	1	4	1	3	6
72	5	4	1	5	2	4	7
73	5	4	1	5	2	4	7
78	5	4	1	5	2	4	7
83	5	4	1	5	2	4	7
84	5	4	1	5	2	4	7
84.2	5	4	1	5	2	4	7
89	5	4	1	5	2	4	7
93	6	5	1	6	1	5	8
94	6	5	1	6	1	5	8
95	6	5	1	6	1	5	8
98	7	5	1	6	1	5	9
100	7	5	1	6	1	5	9
102	7	5	1	6	1	5	9
111	8	6	1	7	3	6	10
133	8	6	1	7	3	6	10
148	8	6	1	7	3	6	10
174	9	6	1	7	3	6	11
198	9	6	1	7	3	6	11
234	9	7	1	2	1	7	12
239	9	7	1	2	1	7	12
249	9	7	1	2	1	7	12
805	10	4	1	7	4	8	13
9653	10	4	1	7	4	8	13
ATCC49782	11	8	1	1	1	9	14
A203	12	8	1	6	1	5	15
BA78	2	2	1	3	1	10	16
GOTA	7	3	1	6	1	11	17
S6	13	4	1	3	2	10	18
S8	13	4	1	3	2	10	18
Ud18	14	9	1	6	1	5	19

**Table 3:** Strains of *U. diversum* isolated from different Brazilian states, São Paulo (SP), Mato Grosso do Sul (MT), Minas Gerais (MG) and Bahia (BA) from 1999 to 2005 and their respective sequence types (ST) and clonal complexes (CC)

Farm	State	Strain	ST	CC
1	SP	A203	ST15	CC4
	SP	59	ST6	CC1
2	SP	GOTA	ST17	CC2
	SP	93	ST8	CC2
	SP	94	ST8	CC2
	SP	95	ST8	CC2
	SP	98	ST9	CC2
	SP	100	ST9	CC2
	SP	102	ST9	CC2
3	SP	72	ST7	SINGLETON
	SP	73	ST7	SINGLETON
	SP	84	ST7	SINGLETON
4	SP	5 T	ST1	SINGLETON
	SP	7 T	ST1	SINGLETON
	SP	10 T	ST1	SINGLETON
	SP	13 T	ST1	SINGLETON
	SP	16 T	ST1	SINGLETON
5	SP	S6	ST18	SINGLETON
	SP	S8	ST18	SINGLETON
6	MT	805	ST13	SINGLETON
	MT	9653	ST13	SINGLETON
7	MG	Ud18	ST19	CC4
8	BA	34	ST2	CC1
	BA	35	ST3	CC1
	BA	37	ST3	CC1
	BA	78	ST7	SINGLETON
	BA	83	ST7	SINGLETON
	BA	84.2	ST7	SINGLETON
	BA	89	ST7	SINGLETON
	BA	BA78	ST16	CC1
9	BA	47	ST3	CC1
	BA	51	ST3	CC1
	BA	52	ST4	CC1
	BA	54	ST5	CC1
	BA	55	ST5	CC1
	BA	56	ST6	CC1
10	BA	234	ST12	SINGLETON
	BA	239	ST12	SINGLETON
	BA	249	ST12	SINGLETON
11	BA	111	ST10	CC3
	BA	133	ST10	CC3
	BA	148	ST10	CC3
	BA	174	ST11	CC3
	BA	198	ST11	CC3

### Phylogeny analyses

**Fig. 2:**
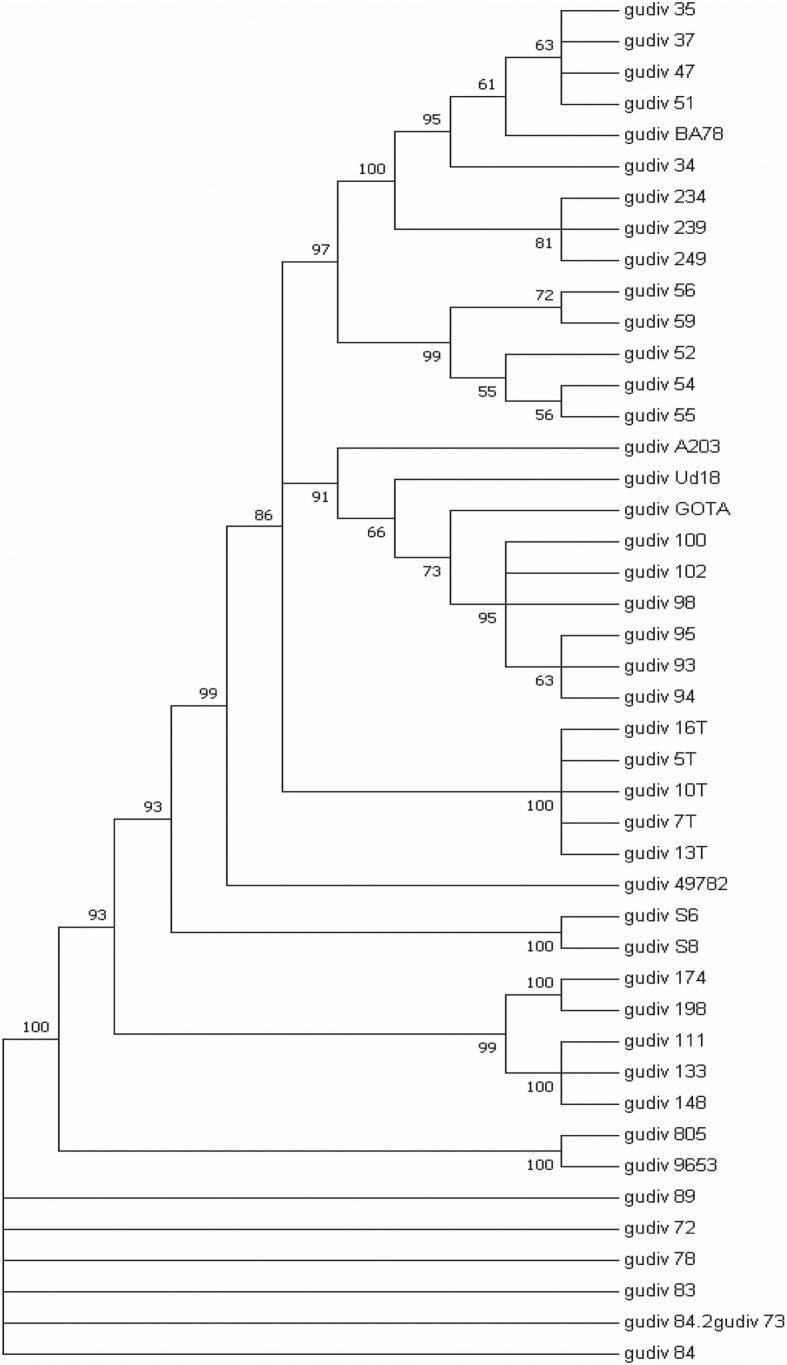
Phylogenetic tree with 45 strains of *U. diversum*, constructed from concatenated sequences of 6 housekeeping genes (*ftsH, rpL22, valS, rpoB, polC and ureA*) using neighbor-joining method 1000 bootstraps

The tree constructed from concatenated sequences of the six housekeeping genes revealed many branches. A variable number of isolates are related in the different branches of the tree, while others are not, which indicates molecular diversity of the strains of *U. diversum*, agreeing with the data obtained by MLST (Fig. 2).

### Virulence factors of *U. diversum*

Detection of the virulence genes was performed for the 45 strains of this study. The size of the amplicons varied between 255 bp and 924 bp (Table 1). Phospholipase D (*pld*) was identified in 18 (40%) isolates, triacylglycerol lipase (*tgl*) in 37 (82.2%), hemolysin (*hlyA*) and MIB (*mib*) found in 35 (77.7%) samples, MIP (*mip*) in 32 (71.1%). The genes *mba* and *VsA* were detected in 33 (73.3%) isolates and the ribose ABC transporter (*tABC*) in 41 (91.1%) ureaplasmas.

### *U. diversum* strains have different profiles of pathogenicity and inflammatory response

**Fig. 3:**
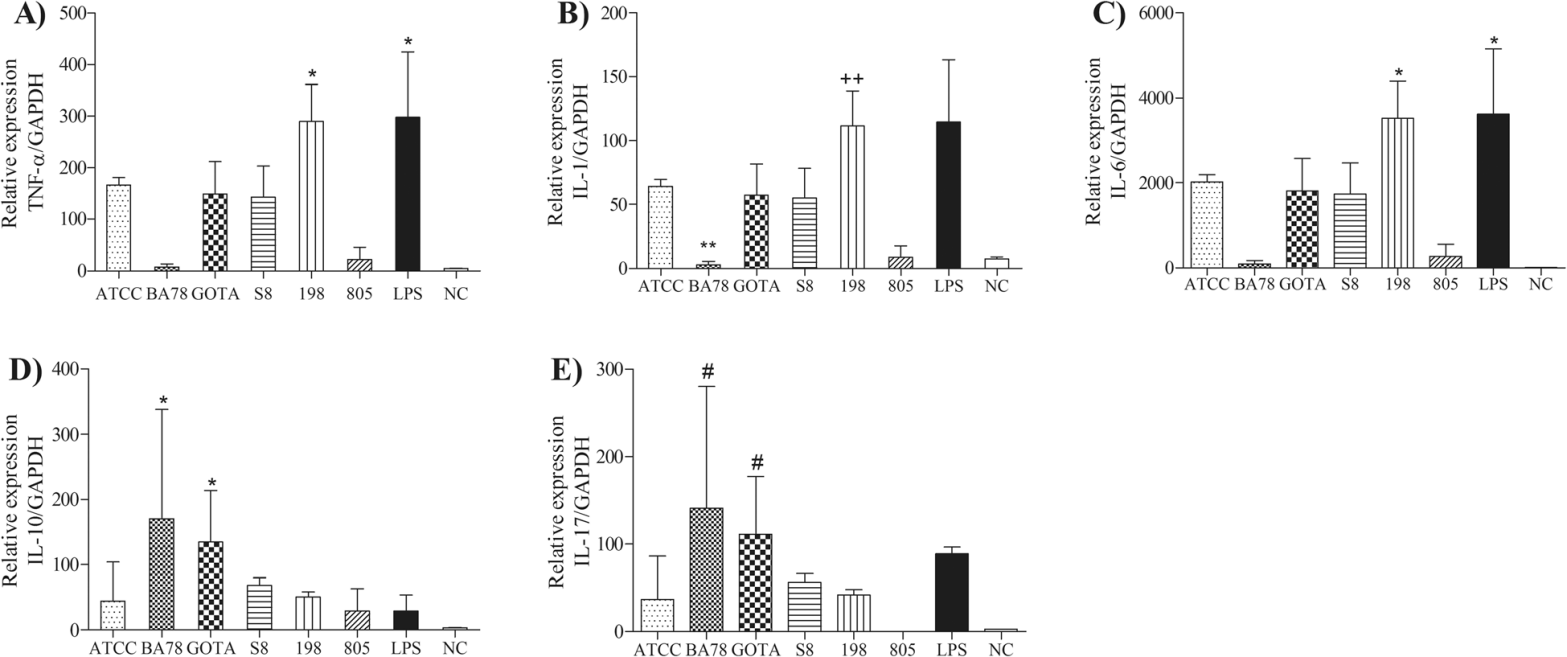
Gene expression of cytokines in bovine monocytes/macrophages infected by 6 strains of *U. diversum* representing different clones and singleton STs: BA78 (CC1), GOTA (CC2), 198 (CC3), ATCC 49782 (CC4), 805 (ST13) and S8 (ST18). **a** TNF expression; **b** IL-1 expression; **c** IL-6 expression; **d** IL-17 expression; **e** IL-10 expression. Groups were compared using the Kruskal-Wallis non-parametric test followed by the Dunn post-test, statistical significance *P* < .05. PBS was used as negative control (NC) and LPS was used as positive control. *Difference with the group NC; **difference with the group LPS; ++ difference with the group BA78; # difference with the group 805

**Fig. 4:**
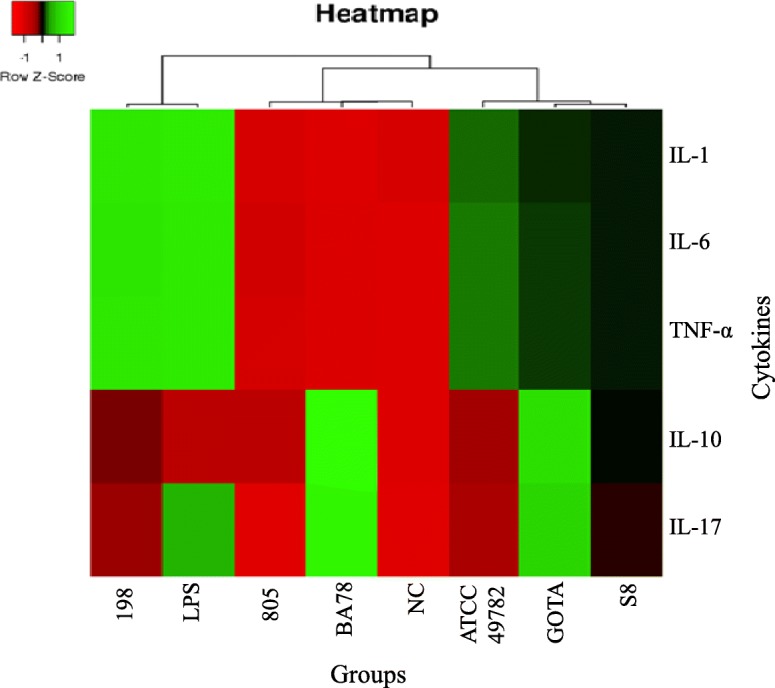
Heatmapper platform formed three clusters according to expression pattern of cytokines TNF-α, IL-1, IL-6, IL-10 and IL-17 in culture of bovine monocytes / macrophages infected by six strains of *U. diversum*: BA78, GOTA, 198, ATCC 49782, 805 and S8, using LPS as positive control and PBS as negative control (NC). Red indicates low expression, and green, high expression of cytokines

**Fig. 5:**
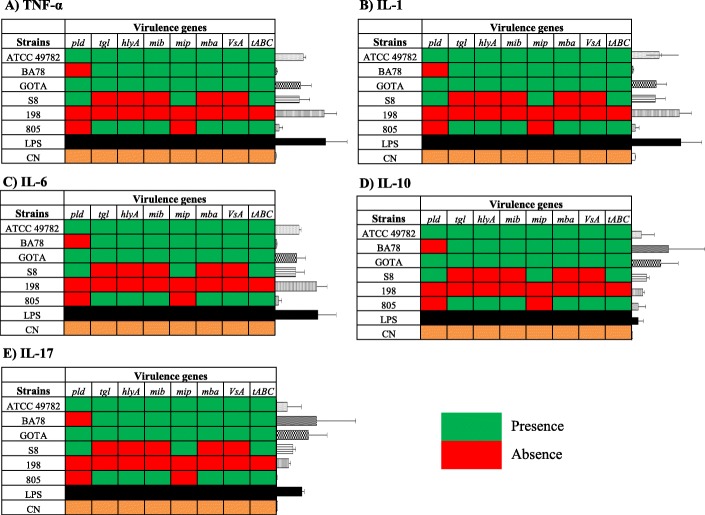
Relation between virulence genes phospholipase D (*pld*), triacylglycerol lipase (*tgl*), hemolysin (*hlyA*), *mib*, *mip*, *mba*, *VsA* and ribose transporter (*tABC*) with gene expression of cytokines in culture of bovine monocytes / macrophages infected by six strains of *U. diversum*, BA78, GOTA, 198, ATCC 4978, 805 and S8. **a**) TNF expression; **b**) IL-1 expression; **c**) IL-6 expression; **d**) IL-10 expression; **e**) IL-17 expression. Green indicates presence of virulence genes, and red indicates a lack of these genes

**Fig. 6:**
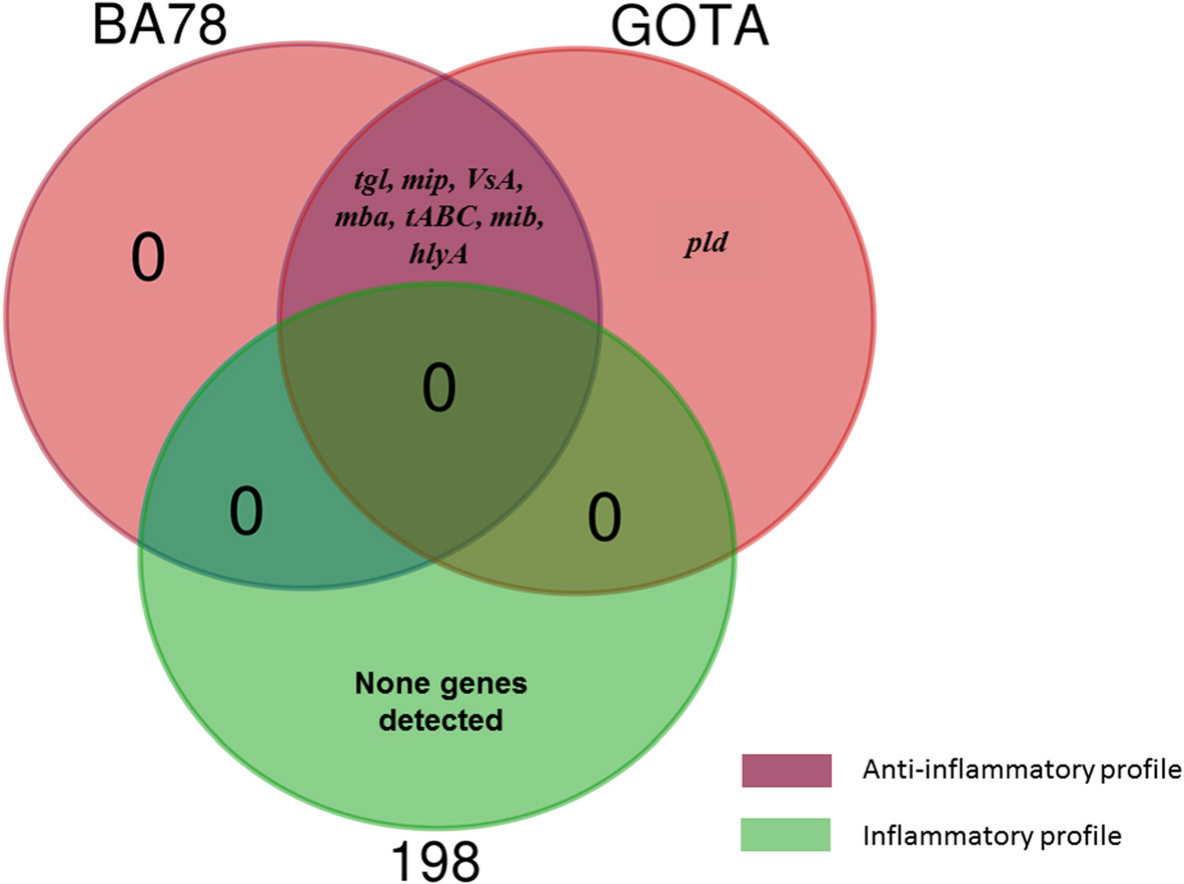
Venn diagram shows virulence genes related to the immune response profile between strains 198, BA78 and GOTA. We observed that the genes shared by BA78 and GOTA induced an anti-inflammatory profile (purple), while strain 198 did not present any gene and induced expression of pro-inflammatory cytokines (green)

Results of cytokine gene expression TNF-α, IL-1, IL-6, IL-10 and IL-17 after infection of monocytes / macrophages by different strains *U. diversum* are shown in Fig. 3. The studied isolates presented different clonal groups and singleton STs: BA78 (CC1), GOTA (CC2), 198 (CC3), ATCC 49782 (CC4), 805 (ST13) and S8 (ST18). For TNF-α and IL-6, it was observed that strain 198 significantly induced the expression of these genes compared to the negative control, and there were differences between the control groups of these cytokines (*P* < 0.05). A statistically significant difference was identified between 198 / BA78 strains and BA78 / LPS expression in IL-1 cytokine expression. For IL-17, there were statistically significant differences between strains BA78 / 805 and 805 / GOTA. And IL-10 gene expression was significantly induced by BA78 and GOTA strains compared to the negative control. Thus, herein, the studied *U. diversum*, showed different cytokine profiles. The isolate 198 induced significant expression of pro-inflammatory cytokines (TNF-α, IL-1 and IL-6), whereas BA78 and GOTA, despite inducing IL-17, led to a significant increase in IL-10. It was observed that, mainly, isolates 198 and BA78 elicited differentiation of the studied immune responses. The heat map separated the cytokine expression profile into three distinct clusters (cluster 1: 198 and LPS; cluster 2: 805, BA78 and NC; cluster 3: ATCC, GOTA and S8) and, together with the qPCR data, shows different expression patterns induced by different strains of *U. diversum* (Fig. 4). The relationship between gene expression of cytokines and virulence genes present in the six selected strains (Fig. 5) was also observed and, in a more summarized way, Table 4 associates the origin of the isolates with pathogenicity and inflammatory modulation (comparing the expression data with the control group), showing that ureaplasmas induce antagonistic profiles of the immune response. Note that the greater the number of virulence genes found, the greater the anti-inflammatory induction (Fig. 6). Thus, while strain 198 (vulvovaginitis origin) did not show any of the virulence genes and increased expression of pro-inflammatory cytokines, strain BA78 (vulvovaginitis origin) induced an anti-inflammatory profile and exhibited most of the evaluated virulence genes.

**Table 4:** Description of the origin of five strains of *U. diversum*, the molecular groups to which they belong, the virulence genes identified and the inflammatory modulation, comparing the gene expression data with the negative control (NC). ↑: increased gene expression

Strains	Source	MLST	Virulence gene found								Modulation (infected x NC)
198	Vulvovaginitis	CC3	Absence of evaluated genes								↑TNF-α, IL-6
S8	Semen	ST18	*pld*		*mip*			*tABC*			None modulation
805	Vulvovaginitis	ST13	*tgl*	*hlyA*	*mib*	*mba*		*VsA*	*tABC*		None modulation
BA78	Vulvovaginitis	CC1	*tgl*	*hlyA*	*mib*	*mip*	*mba*	*VsA*	*tABC*		**↑**IL-10
GOTA	Vulvovaginitis	CC2	*pld*	*tgl*	*hlyA*	*mib*	*mip*	*mba*	*VsA*	*tABC*	**↑**IL-10

## Discussion

The diversity of strains of *U. diversum* was analyzed through an MLST technique. Based on previous studies, in which genes were chosen for their chromosomal location and diversity of sequences found, [9] and the Manatee database (ATCC strain 49,782), a set of housekeeping genes was selected: *ftsH, rpL22*, *valS, thrS, rpoB, polC* and *ureA.* However, *thrS* was not properly sequenced*.* The *ftsH* gene encodes essential proteases [10], *rpL22* encodes ribosomal proteins [11], *polC* encodes the unique DNA polymerase found in mycoplasmas, DNA polymerase III [12], *rpoB* encodes β-subunit of RNA polymerase [13], *valS* catalyzes amino acid activation and tRNA transfers [14] and the *ureA* gene encodes urease that catalyzes urea in ammonia [15]. Out of 6 genes evaluated, *rpL22* did not present polymorphisms between the strains evaluated, which does not reduce the sensitivity and efficiency of the method and shows the possibility of executing the MLST scheme with five housekeeping genes for molecular typing of *U. diversum*, as was done for *M. agalactiae* [16], *M. bovis* [17], for example. In addition, it is interesting to develop an MLST scheme combining housekeeping and virulence genes (expanded multilocus sequence typing - eMLST) to improve the high resolution of the technique [18] and allow the identification of important molecular markers for the differentiation of pathogenic and non-pathogenic strains.

The MLST scheme has already been used for mycoplasmas: *M. arginini* [19], *M. pneumoniae* [20], *M. hyorhinis* [21], *M. agalactiae* [22], *M. hyopneumoniae* [23], *M. mycoides* [24], *M. bovis* [25]. In 2013, MLST technique was developed for species of ureaplasmas that infect humans [9]. The present study is the first report of MLST applied to the diversity analysis of *U. diversum* and identified 19 STs. Different genetic sequences are classified as distinct alleles. The set of the alleles form the allelic profile and defines the ST, assigning a random number [26]. In this study ST7 was the most frequent; however, it was not grouped in CC. From the ureaplasma studied, 42.3% were singleton STs. These STs did not share, at least, three alleles with other STs, and therefore are considered the most genetically diverse isolates [26]. The variety of STs found indicates that there was a gene recombination [27].

Clonal complexes are formed, at least, from two different STs that must share, at least, three alleles with each other [19]. Thus, we detected four CCs: CC1, CC2, CC3, and CC4, indicating a genetic variation among isolates. These data suggest that herd and animal management may influence ureaplasma variability due to environmental pressures [26]. In addition, the absence of some DNA repair mechanisms helps preserve the genetic diversity of mollicutes [28]. Phylogenetic data also expressed this molecular diversity observed among strains of *U. diversum*. Some isolates were clustered while others were not, according to the data obtained by MLST. Isolates from the same clade are equivalent to isolates belonging to the same clone or the same ST; in addition, non-grouped strains also represent ST singletons (Fig. 2).

Virulence factors were also analyzed for the studied *U. diversum*. Based on the ATCC strain sequencing [2], primers were designed for different genes. We observed that the phospholipase D gene (*pld*) was found in 40% of the isolates. This enzyme alters the synthesis of prostaglandins E_2_ and F_2a_ by endometrial cells, rendering pregnancy difficult [3]. Another enzyme from the lipase family, triacylglycerol lipase, was identified in 82.2% of the isolates. These enzymes are excreted and damage host tissues [29]. The hemolysin gene, detected in 77.7% of the isolates, causes erythrocyte hemolysis and has 63.1% identity with the hemolysin protein sequence found in human ureaplasmas [2]. The MIB-MIP system has been described recently; MIB is a protein that binds strongly to IgG, whereas MIP cleaves IgG, contributing to immune system evasion [30]. We reported that 77.7% of the isolates had *mib*, and 71.1% had *mip*. The surface molecules were also observed; *mba* and *VsA* were detected in 73.3% of the isolates. MBA are antibody targets, stimulate monocytes / macrophages and have cytolytic activity, whereas VsA is critical for bacterial survival due to antigenic variations [31]. A ribose transporter gene (*tABC*) was identified in 91.1% of ureaplasma. These sugars are captured by *U. diversum* and are incorporated to form a polysaccharide capsule [2].

To evaluate the implication of the different clones found under the cytokine gene expression patterns, the culture of bovine monocytes / macrophages was infected with six different strains of *U. diversum* (one strain from each CC and two singleton STs) previously selected. We observed that strain 198 significantly increased the expression of the pro-inflammatory cytokines TNF-α and IL-6 compared to the control, as well as induced the expression of IL-1 compared to strain BA78. Cytokine IL-17 was induced by strains BA78 and GOTA, in relation to isolate 805. Therefore, these data show different patterns in inducing pro-inflammatory cytokines by different strains of *U. diversum*. Other literature data also demonstrate cytokine induction by different strains of a microorganism, such as a study that showed differences in the levels of TNF-α, IL-6 and IL-17 induced by different strains of *E. coli* in bovine breast tissue [7] or increased TNF-α expression in bovine PBMC infected with *M. bovis*, as well as IL-6, IL-10 and TNF-α expression induced by culture supernatant of this bacterium [32]. Regarding anti-inflammatory cytokine expression, our results show the induction of IL-10 by BA78 and GOTA strains compared to untreated cells (*P* < 0.05). In this context, it was observed that bovine monocytes infected with *M. bovis* considerably increase the secretion of IL-10 by negatively regulating pro-inflammatory cytokines [33]. Note that BA78 and GOTA strains induced the cytokine expression of an antagonistic nature; therefore, IL-17 expression did not prevent an anti-inflammatory modulation. This cytokine produced by Th17 cells recruits neutrophils; however, it has been reported that IL-17 does not increase the survival of bovine neutrophils after infection with *M. bovis* [34] and the need for synergy of IL-17 with other cytokines has been proposed for improving its function [35].

Regarding data of the genic expression to virulence of the strains, it was observed that 198 induced an inflammatory profile, but did not exhibit any virulence gene, whereas BA78 induced an anti-inflammatory profile and exhibited all the genes, except for *pld*. Inflammation results from the recognition of lipid-associated membrane proteins (LAMPS) through TLRs 2 and 4 that activate the MyD88 and NF-KB-signaling pathway [36, 37]. However, mycoplasma infections are not necessarily related to strong inflammatory responses, because LAMPS can suppress inflammation by inducing IL-10 production [33]. In addition, altered surface proteins, such as VsA and MBA, make it difficult to recognize bacteria through the immune cell [31, 38]. Thus, it is suggested that the BA78 strain used its virulence factors to evade immunity and promote its survival [33], whereas strain 198 may contain other lipoproteins (not evaluated in this study) that were recognized and induced the expression of pro-inflammatory cytokines. Further studies should be conducted to better understand the role of virulence genes in *U. diversum* and their relation with the immune response.

## Conclusion

The genetic variability between strains of *U. diversum* is poorly understood in cattle. Our findings contributed to the understanding of *U. diversum* variability using MLST, a tool that showed intraspecies diversity of the 45 isolates evaluated, assigning them different STs and 4 CCs. The results of gene expression demonstrated differences in the immune response against different strains, mainly between strains 198 and BA78 that modulate inflammatory and anti-inflammatory profiles, respectively. The isolates also exhibited differences in virulence factors. Thus, this study demonstrated the existence of different clones with a consequent variation in virulent determinants that may be associated with gene expression, and these different patterns can impact the pathogenicity of ureplasmas, which could be important to consider in future studies.

## Methods

### Cultures of *U. diversum*

The strain of *U. diversum* ATCC 49782 and 44 field isolates were supplied by the Mycoplasma Laboratory of the Biomedical Sciences Institute, University of São Paulo. Some strains were isolated from cows with granulomatous vulvovaginitis, and others were isolated from the semen of healthy bulls. The bacteria were obtained from the following four states: 19 isolates in São Paulo (farms 1, 2, 3, 4 and 5), 2 isolates from Mato Grosso do Sul (farm 6), 1 isolate from Minas Gerais (farm 7) and 22 isolates from Bahia (farms 8, 9, 10, 11) (Figure S1 in Supplementary file). All microorganisms were cultured in 5 mL of UB broth at 37 °C for 2 days [2]. After growth, the cultures were stored at − 80 °C for the study. The isolates were first submitted to genetic analysis and, after the genetic groups were defined, and representative profiles of each group were evaluated for pathogenicity.

### DNA extraction

DNA extraction was performed using the Nucleospin DNA-tissue commercial kit (Macherey-Nagel, Düren, Germany, 2016), following the manufacturer’s recommendations.

### Primers design for MLST and virulence genes

Based on descriptions from the literature, seven housekeeping genes were selected for the MLST scheme - *ftsH, rpL22, valS, thrS, rpoB, polC* [9] and the *ureA* gene [2] - while other genes were selected for virulence analysis - phospholipase D (*pld*) (gudiv_472), triacylglycerol lipase (*tgl*) (gudiv_748), hemolysin (*hlyA*) (gudiv_91), MIB-MIP system (*mib,mip*) (gudiv_161/gudiv_162), surface molecules, such as multiple banded antigens (*mba*) (gudiv_653), and variable surface antigen lipoprotein (*VsA*) (gudiv_179) and ribose ABC transporter (*tABC*) (gudiv_307) [2]. After selection of loci for MLST and virulence factors, primers were designed. Through the Manatee database (https://manatee.igs.umaryland.edu) it was possible to access the categorized genes of *U. diversum,* strain ATCC 49782. Genomic sequences of interest were obtained and possible primer sequences were chosen at random and their analyses were started through the sites https://www.idtdna.com/calc/analyzer and https://www.bioinformatics.org/sms/rev_comp.html. After checking important parameters for constructing primers (size, GC content, melting temperature and annealing, formation of secondary structures), the similarity performed by BLAST was validated (https://blast.ncbi.nlm.nih.gov/Blast.cgi) to confirm the specificity of the primers for *U. diversum*. Forward and reverse primers for each of the selected genes (housekeeping and virulence) are described in Table 1.

### Polymerase chain reaction and electrophoresis

Amplifications were performed with a total volume of 25 μL, with 1 μL of DNA, 10x PCR buffer (10 mM Tris-HCl, pH 9.0, 50 mM KCl), 1.5 mM MgCl_2_; 200 μM dNTP, 50 pmol of each primer and 1.5 U of Taq DNA polymerase (Invitrogen®, Brazil). The reactions for *ftsH, polC, rpL22, urea, valS* and all virulence genes occurred with initial denaturation of 94 °C for 5 min followed by 35 thermal cycles each consisting of 94 °C for 30 s, 54 °C for 30 s and 72 °C for 1 min, concluding with a final extension at 72 °C for 5 min. While for the *rpoB* and *thrS* genes, there was initial denaturation of 94 °C for 5 min, followed by 35 thermal cycles each consisting of 94 °C for 30 s, 50 °C for 30 s and 72 °C for 1 min, concluding with a final extension of 72 °C for 5 min. The targeted products were analyzed by 2% agarose gel electrophoresis, stained with 2.5 μL ethidium bromide (10 mg / mL), visualized and photographed under UV light. A marker was used as standard to evaluate the size of amplified fragments - 100 bp DNA Ladder (Invitrogen®, Brazil).

### PCR product purification and sequencing for MLST technique

After electrophoresis, the total volume of the remaining PCR reaction was precipitated with 500 μL of 65% isopropanol, followed by centrifugation at 19,000 xg for 5 min, washing with 250 μL of 70% alcohol and further centrifugation at 19,000 xg for 5 min. The tubes were inverted for 1 h to dry at room temperature, then resuspended with 20 μL ultrapure water and the primers diluted to 5 pmol. Sanger sequencing reactions were performed at the Biomedical Sciences Institute, Federal University of Sao Paulo according to the protocol for the MegaBACE 1000, using the DYEnamic ET Dye Terminator Kit (with Thermo Sequenase™ II DNA Polymerase) code US81090. The sequences obtained were analyzed by Sequence Analyzer software using the Cimarron Caller Base 3.12.

### Allele, sequence type, and clonal complex assignment

The sequencing data were analyzed using nonredundant database (NRDB) comparison tools (https://pubmlst.org/analysis) to assign alleles. Distinct alleles were identified by comparing the sequences of the same gene, and these were assigned arbitrary numbers. The combination of the seven identified alleles formed the allelic profile, which was used to determine the sequence type (ST), and assigned an arbitrary number. Each observed variation, of at least one allele, generated a new ST [9, 26]. Clonal complexes (CC) were formed by different STs that shared, at least, three loci with one other member in the group. A clonal complex must include, at least, two STs to be defined as a new clonal group. STs that were not in any CC, were ST singletons [26].

### Phylogenetic analysis

The phylogenetic tree was constructed by Molecular Evolutionary Genetics Analysis 6 (MEGA) using the neighbor-joining method with 1000 bootstraps from the concatenated sequences of housekeeping genes. The sequence was concatenated using UNION, a tool of the European Molecular Biology Open Software Suite (EMBOSS), (http://www.bioinformatics.nl/emboss-explorer/). Then, the sequence was aligned by MAFFT 7 [2].

### Isolation of bovine monocytes / macrophages

Fifty milliliters of peripheral blood was collected from the abdominal vein of a cow in EDTA vacutainer tubes and diluted 1:1 in PBS (pH 7.4) [32]. Then, 10 mL of blood was added to 3 mL of Ficoll-Histopaque (density: 10771 g/mL, Sigma-Aldrich, Brazil), centrifuged at 400 xg at 4 °C for 20 min, and the layer of peripheral blood mononuclear cells (PBMC) was removed, washed in PBS 1X and centrifuged again at 400 xg for 10 min. For monocyte isolation, PBMC was resuspended in solution A (5 mL of RPMI 1640 medium containing 10% fetal bovine serum) and mixed in 5 mL solution B (5 mL RPMI 1640 medium + 4.75 mL Percoll + 0.325 mL 10X PBS). This suspension was centrifuged at 400 xg for 30 min at 20 °C and monocytes were removed between the interface of both solutions. To evaluate cell viability, 0.1% Trypan Blue (viability > 90%) was used; the cells were counted in a Neubauer chamber, adjusted concentration 4 x 10^5^cells / mL and grown in a 5% CO_2_ chamber at 37 °C. After 24 h, the monocytes / macrophages were infected.

### Preparation of inoculum and infection of bovine monocytes / macrophages by *U. diversum*

Six strains (ATCC 49782, BA78, GOTA, S8, 198 and 805), representative of different genetic groups, were grown in UB broth, centrifuged at 300 xg for 30 min, resuspended in PBS and the culture suspension was quantified in 96-well microplates to obtain the inoculum based on a Colorimetric Change Unit (CCU). Monocytes / macrophages (4 x 10^5^cells / mL) were infected with 10^5^ ureaplasma / mL for GOTA, 198, 805 and 10^4^ ureaplasma / mL for ATCC 49782, BA78 and S8. Cells treated with LPS representing the positive control and PBS were added to the cells as negative control. After 6 h of infection, the supernatant was removed, 50 μL of trypsin was added to the wells, followed by 50 μL of fetal bovine serum. Finally, the cells were resuspended in 200 μL of RNAlater™ (Invitrogen, Brazil) and stored at − 80 °C for later use.

### Gene expression

The mRNA of cells infected with ureaplasma was extracted according to manufacturer’s instructions for PicoPure kit (Applied Biosystems, Brazil), with DNAse treatment and RNA elution in a 11 μL kit elution solution. The cDNA was obtained by reverse transcription (RT) from the mRNA, using the SuperScript® III Reverse Transcriptase kit (Applied Biosystems, Brazil). The cDNA was used in a custom StepOnePlus Real-Time PCR System (Applied Biosystems, Brazil) with SYBR Green (Qiagen-SABioscience, Brazil) to determine the gene expression of TNF-α, IL-1β, IL-6, IL − 10 and IL-17 (Qiagen-SABioscience, Brazil), following the cycle: 50 °C for 10 min; 95 °C for 10 min; and 45 cycles of denaturation at 95 °C for 15 s, annealing at 60 °C for 1 min. The melting curve was evaluated at the end of the reaction to observe the amplification specificity. The data was analyzed using the 2^-∆∆CT^ [39]. Standardization was performed based on GAPDH expression.

### Expression heat map

The relative expression data of the TNF-α, IL-1β, IL-6, IL-10 and IL-17 cytokines, induced by different strains of *U. diversum*, were analyzed using the heatmapper platform (http: // www. Heatmapper.ca/expression/) and represented as a heatmap. The unsupervised hierarchical grouping was performed using the average distance and the Euclidean distance as metrics.

### Statistical analysis

In this study, three independent experiments were carried out for each of the six strains of *U. diversum*. To compare cytokine gene expression of different strains, the Kruskal-Wallis non-parametric test was used, followed by Dunn’s post-hoc test, which makes paired comparisons. All analyses were performed using the GraphPad-Prism 6.0 software (GraphPad software, San Diego, CA-USA). Statistically significant differences were found with *P* values equal to or less than 0.05, using IC 95% for relative expression of cytokines for the different strains.

## Supplementary information

**Additional file 1: Figure S1.** The strains were isolated from different cattle herds distributed in four states highlighted on the map of Brazil: São Paulo (SP), Mato Grosso do Sul (MT), Minas Gerais (MG) and Bahia (BA) with their respective STs.

## Data Availability

The DNA sequences generated and/or analysed during the current study are available in the GenBank repository, Accession: PRJNA632017. The others datasets used and/or analyzed during the current study are available from the corresponding author on reasonable request.

## Abbreviations

ATCC: American Type Culture Collection
CC: Clonal complexes
EDTA: Ethylenediamine tetraacetic acid
EMBOSS: European Molecular Biology Open Software Suite
*ftsH*: ATP-dependent zinc metalloprotease
GAPDH: Glyceraldehyde-3-Phosphate Dehydrogenase
GC: Guanine-cytosine content
*hlyA*: Hemolysin
IgG: Immunoglobulin G
IL-1: Interleukin-1
IL-10: Interleukin-10
IL-17: Interleukin-17
IL-6: Interleukin-6
LAMPS: Lipid-associated membrane proteins
MAFFT 7: Multiple alignment program for amino acid or nucleotide sequences
MBA/*mba*: Multiple Banded Antigen
*mib,mip*: MIB-MIP system
MLST: Multilocus sequence typing
NRDB: Nonredundant database
PBS: Phosphate buffered saline
PCR: Polymerase Chain Reaction
PBMC: Peripheral blood mononuclear cells
*pld*: Phospholipase D
*polC*: DNA polymerase III
tABC: Ribose transporter
*rpL22*: Ribosomal Protein L22
RPMI: Roswell Park Memorial Institute
*rpoB*: RNA polymerase beta-subunit
ST: Sequence types
TNF-α: Tumor Necrosis Factor alpha
*tgl*: Triacylglycerol lipase
UB: Ureaplasma base
*ureA*: Urease subunit alpha
*valS*: Valine-tRNA ligase
VsA: Variable surface antigens
dNTP: Deoxynucleotides.
