# Supplementary figures and images for: Multilocus sequence typing characterizes diversity of Ureaplasma diversum strains, and intra-species variability induces different immune response profiles
Source: BMC Vet Res. 2020 May 26;16:163. doi: 10.1186/s12917-020-02380-w (PMC7249313; doi:10.1186/s12917-020-02380-w)

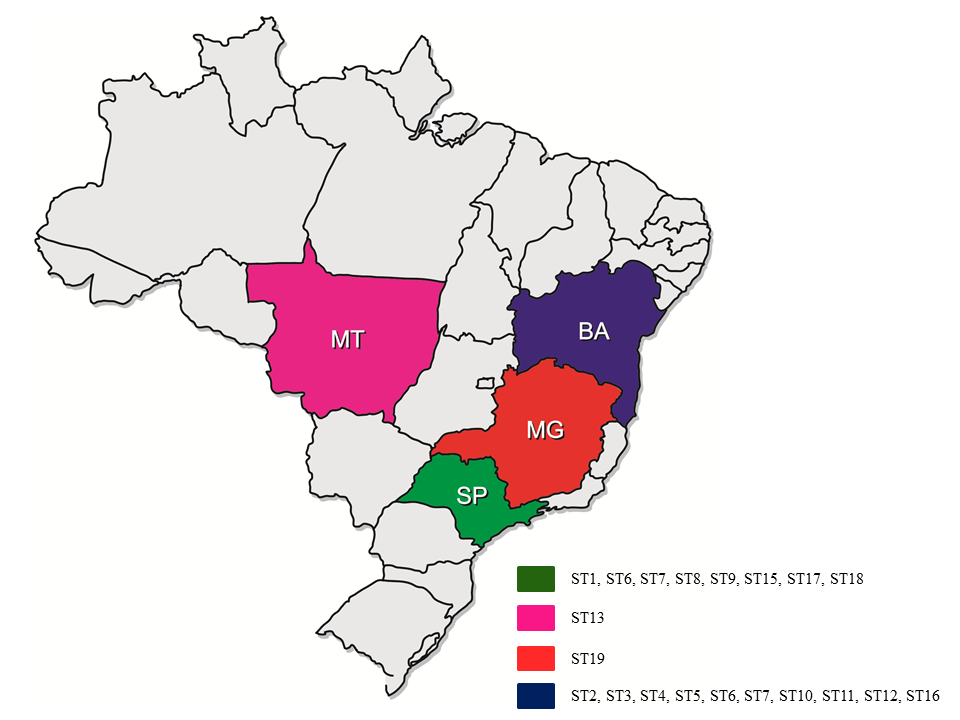

Supplement: Supplementary file 1 — Additional file 1: Figure S1. The strains were isolated from different cattle herds distributed in four states highlighted on the map of Brazil: São Paulo (SP), Mato Grosso do Sul (MT), Minas Gerais (MG) and Bahia (BA) with their respective STs. [file 12917_2020_2380_MOESM1_ESM.tif]
